# Differential Role of Pontomedullary Glutamatergic Neuronal Populations in Sleep-Wake Control

**DOI:** 10.3389/fnins.2019.00755

**Published:** 2019-07-30

**Authors:** Evelyn T. M. Erickson, Loris L. Ferrari, Heinrich S. Gompf, Christelle Anaclet

**Affiliations:** Department of Neurobiology, University of Massachusetts Medical School, Worcester, MA, United States

**Keywords:** neuronal circuitry, DREADDs, brainstem, parafacial zone, sleep-wake control, sublaterodorsal nucleus, parabrachial nucleus

## Abstract

Parafacial zone (PZ) GABAergic neurons play a major role in slow-wave-sleep (SWS), also called non-rapid eye movement (NREM) sleep. The PZ also contains glutamatergic neurons expressing the vesicular transporter for glutamate, isoform 2 (Vglut2). We hypothesized that PZ Vglut2-expressing (PZ^Vglut2^) neurons are also involved in sleep control, playing a synergistic role with PZ GABAergic neurons. To test this hypothesis, we specifically activated PZ^Vglut2^ neurons using the excitatory chemogenetic receptor hM3Dq. Anatomical inspection of the injection sites revealed hM3Dq transfection in PZ, parabrachial nucleus (PB), sublaterodorsal nucleus (SLD) or various combinations of these three brain areas. Consistent with the known wake- and REM sleep-promoting role of PB and SLD, respectively, chemogenetic activation of PB^Vglut2^ or SLD^Vglut2^ resulted in wake or REM sleep enhancement. Chemogenetic activation of PZ^Vglut2^ neurons did not affect sleep-wake phenotype during the mouse active period but increased wakefulness and REM sleep, similar to PB^Vglut2^ and SLD^Vglut2^ activation, during the rest period. To definitively confirm the role of PZ^Vglut2^ neurons, we used a specific marker for PZ^Vglut2^ neurons, Phox2B. Chemogenetic activation of PZ^Phox2B^ neurons did not affect sleep-wake phenotype, indicating that PZ glutamatergic neurons are not sufficient to affect sleep-wake cycle. These results indicate that PZ glutamatergic neurons are not involved in sleep-wake control.

## Introduction

Over the past few years, the medullary parafacial zone (PZ) has been identified as a strong sleep-promoting brain area (Anaclet and Fuller, 2017). Both disruption of PZ GABAergic transmission (Anaclet et al., 2012) and chemogenetic inhibition of PZ GABAergic (PZ^*GABA*^) neurons (Anaclet et al., 2014) result in insomnia. More importantly, chemogenetic activation of PZ^GABA^ neurons strongly increases SWS amount and consolidation and enhances cortical EEG slow-wave activity (SWA), a marker of SWS depth (Anaclet et al., 2014). Finally, chemogenetic activation of PZ^GABA^ neurons counteracts the wake-promoting action of psychostimulants (Anaclet et al., 2018). The PZ is generally located dorsal and lateral from the facial nerve but its exact boundaries are not precisely defined. A recent study has shown that, in mouse, the parvicellular reticular nucleus part alpha (PCRtA), ventral from PZ, does not contain sleep-active neurons (Sakai, 2017), indicating that the PZ sleep promoting neuronal population does not include the PCRtA.

In rats, about 40% of PZ neurons are sleep-active (Alam et al., 2018) and cell body specific PZ lesions result in insomnia (Anaclet et al., 2012). More specifically, PZ^GABA^ are involved in slow-wave-sleep (SWS) control. However, cFos expression, a marker of neuronal activity, showed that about half of sleep-active neurons are GABAergic (Anaclet et al., 2012), indicating that in the PZ, non-GABAergic neurons are also involved in sleep control. Within the PZ, the only other known neuronal population is glutamatergic, expressing the vesicular glutamate transporter isoform 2 (Vglut2; *in situ* hybridization data are available in Allen Mouse Brain Atlas, Allen Institute for Brain Science^1^). We hypothesized that PZ glutamatergic neurons (PZ^Vglut2^) are also involved in sleep control and act synergistically with sleep-active PZ^*GABA*^ neurons to promote SWS. To start testing this hypothesis, we chemogenetically activated PZ^Vglut2^ neurons. Specific targeting of PZ was challenging and sleep phenotypes were difficult to interpret due to the possible transfection of the neighboring parabrachial (PB) and sublaterodorsal (SLD) nuclei that are involved in wakefulness and rapid eye movement (REM) sleep, respectively (Clement et al., 2011; Fuller et al., 2011). To get around these obstacles and specifically test the involvement of PZ glutamatergic neurons in sleep-wake control, we used Phox2B, a transcription factor expressed in PZ but not PB or SLD glutamatergic neurons. Data indicate that PZ glutamatergic neurons are not involved in sleep-wake control. Additionally, we found that excitation of PB or SLD glutamatergic neurons promotes wakefulness or REM sleep, respectively, results that are complimentary to the reduction in wakefulness or REM sleep previously observed following lesion of PB or SLD, respectively (Clement et al., 2011; Fuller et al., 2011).

## Materials and Methods

### Animals

In order to visualize Vglut2-, Phox2B- and Vgat-expressing neurons, Vglut2-IRES-cre [Jackson Laboratory #016963 (Vong et al., 2011)], Phox2B-IRES-cre [Jackson Laboratory #016223 (Rossi et al., 2011)] and Vgat-IRES-cre [Jackson Laboratory #016962 (Vong et al., 2011)] mice were crossed with a cre-dependent reporter mouse Flox-L10-GFP [Jackson Laboratory #24750 (Liu et al., 2014)], producing Vglut2-GFP, Phox2B-GFP and Vgat-GFP mouse lines. Thirty two adult male Vglut2-GFP mice, seven adult male Phox2B-GFP and one adult male Vgat-GFP (8–12 weeks, 20–25 g) mice were used in this study. Mice were bred at our animal facility and underwent genotyping both before and after experiments. All procedures were approved by the Institutional Animal Care and Use Committee of Beth Israel Deaconess Medical Center and of University of Massachusetts Medical School.

### Surgery

Naïve mice were anesthetized with ketamine/xylazine [100 and 10 mg/kg, respectively, intraperitoneal (IP)] and then placed in a stereotaxic apparatus. To selectively express the hM3Dq receptors in glutamatergic (Vglut2+) or Phox2B-expressing neurons of the PZ, we performed bilateral injections of an adeno-associated viral (AAV; serotype 10) vector expressing the hM3Dq receptor in a cre-dependent configuration [hSyn-DIO-hM3Dq-mCherry-AAV; (Anaclet et al., 2014)] into the PZ [coordinates from Bregma: Antero-posterior = −5.6 mm, Lateral = ± 1.0 mm, Dorso-ventral = −4.2 mm, as per the mouse atlas of Paxinos and Watson (Paxinos and Franklin, 2001)] of *Vglut2-IRES-cre* (PZ^Vglut2–hM3Dq^) mice, *Phox2B-IRES-cre* (PZ^Phox2B–hM3Dq^) mice or non-cre expressing littermate control mice. Injections of the viral vector (60 nl) into the PZ of these mice were performed using a compressed air delivery system as previously described (Anaclet et al., 2010). After injections, mice were implanted with four EEG screw electrodes (Pinnacle Technology Inc., Catalog #8403) and two flexible electromyogram (EMG) wire electrodes (Plastics One, catalog #E363/76/SPC), previously soldered to a 6-pin connector (Heilind Electronics, catalog #853-43-006-10-001000) and the assembly was secured with dental cement. The scalp wound was closed with surgical sutures and the mouse was kept in a warm environment until resuming normal activity as previously described (Anaclet et al., 2015).

### Sleep-Wake Monitoring

Three weeks after surgery, the mice were housed individually in transparent barrels in an insulated sound-proofed recording chamber maintained at an ambient temperature of 22 ± 1°C and on a 12 h light/dark cycle (lights-on at 7 A.M., Zeitgeber time: ZT0) with food and water available *ad libitum*. Mice were habituated to the recording cable for 5–7 days before starting polygraphic recording. Cortical EEG (ipsilateral fronto-parietal leads) and EMG signals were amplified (A-M System 3500, United States) and digitalized with a resolution of 500 Hz using Vital Recorder (Kissei, Japan). Mice were recorded for a 24 h baseline period followed by IP injections of saline (control injection) or Clozapine-N-oxide (CNO, NIMH Chemical Synthesis and Drug Supply Program; 0.3 mg/kg in saline). Injections were performed at 10 A.M. (10:00, ZT3, light period, time of high sleep-drive) and 7 P.M. (19:00, ZT12, beginning of the dark period, time of high wake-drive), in a randomized cross-over design, with each injection separated by a 2–3 day washout period. In each experiment, recordings were simultaneously made from an equal number (batches of 2–4) of PZ^Vglut2–hM3Dq^ and PZ^Vglut2–wt^ mice.

### Sleep Scoring and Analysis

Using SleepSign for Animal (Kissei, Japan) assisted by spectral analysis using fast Fourier transform, polygraphic records were visually scored in 10 s epochs for wakefulness (W), SWS, and REM sleep. The percentage of time spent in wake, SWS and REM sleep were summarized for each group and each condition. The SWS to REM sleep latency is defined as the time between the onset of the first SWS episode, lasting >20 s, after injection and the onset of the first REM sleep episode, lasting >10 s.

Sleep-wake fragmentation was assessed by analyzing the distribution of each vigilance stage in different bout lengths. Vigilance stages were separated into eight bout lengths (<30, 40–70, 80–150, 160–310, 320–630, 640–1270, 1280–2550, and >2550 s) (Mochizuki et al., 2004; Kantor et al., 2013). For each vigilance stage, the number of episodes and the percentage of the vigilance stages occurring in each bout length were used to produce a time-weighted frequency histogram.

Recordings were scored again in 5 s epochs to allow for performance of an EEG power spectrum analysis. On the basis of visual and spectral analysis, epochs containing artifacts occurring during active wake (with large movements) or containing two vigilance states were visually identified and omitted from the spectral analysis. Recordings containing wake artifacts during more than 20% of the time were removed from the spectral analysis. EEG power spectra were computed for consecutive 5 s epochs within the frequency range of 0.5–120 Hz using a fast Fourier transform routine (FFT). The data were collapsed into 0.5 Hz bins. To determine the effect of injection on sleep-wake power spectra, EEG power spectra were analyzed during the 3 h period of time post-injection, starting 10 min after injection as a previous study had shown that CNO injection significantly affected SWS amount during 3 h post-injection and SWS latency was no more than 10 min (Anaclet et al., 2014). The data were standardized by expressing each frequency bin as a percentage relative to the same bin under baseline conditions from the same mouse and from the same time of the day (same Zeitgeber time). To analyze the EEG frequency bands, power bins were summed in δ 0.5–5 Hz, θ 5–9 Hz, α 9–15 Hz, β 15–30 Hz, low γ 30–60 Hz and high γ 60–120 Hz, and expressed in percentage of baseline power band, from the same circadian time.

Statistical analysis was performed using Prism v6 (GraphPad Software, San Diego, CA, United States). Following confirmation that the data met the assumptions of the ANOVA model, two-way ANOVA followed by a *post hoc* Bonferroni test were used to compare the effects of the drug injection and time period on sleep-wake parameters, the effect of the drug injection and the distribution of vigilance episodes, or the effect of drug injection and power band on cortical EEG power density. Paired Student’s *t*-test was used to compare the effects of the drug injection on SWS to REM sleep latency. Sample size and power calculations were performed *post hoc* at http://www.biomath.info, using means and standard deviations derived from our analysis. The present study was sufficiently powered to detect effect sizes.

### Immunostaining and RNAscope

At the end of the behavioral experiments, mice were deeply anesthetized with ketamine/xylazine (200 and 20 mg/kg, respectively) and perfused transcardially with 20 ml of saline, followed by 100 ml of neutral phosphate-buffered formalin (4%; Thermo Fisher Scientific). Brains were removed from the skull and incubated in neutral phosphate-buffered formalin (4%; Thermo Fisher Scientific) for 2 h, followed by 20% sucrose until they sank.

**FIGURE 1:**
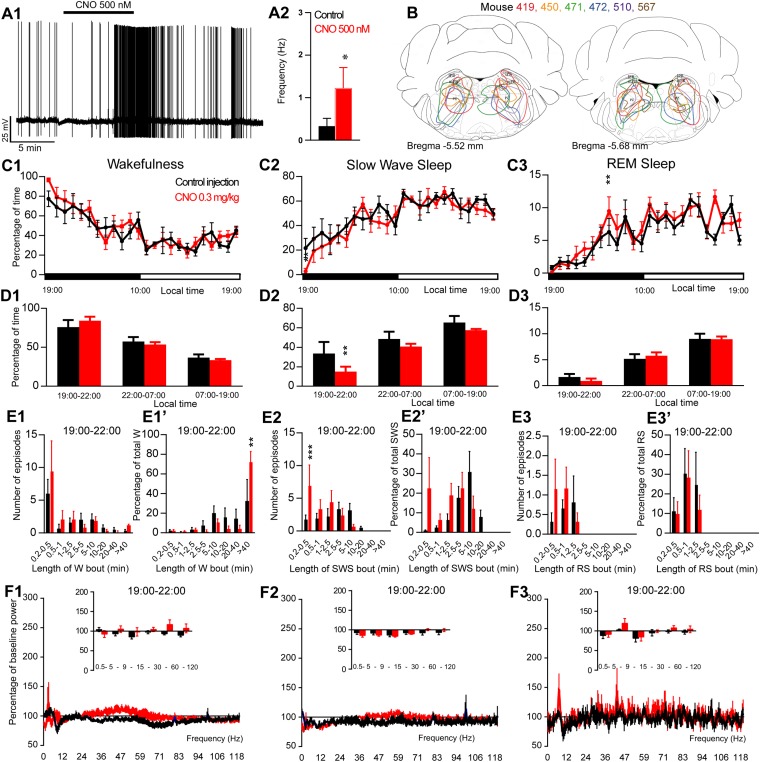
Activation of PZ glutamatergic neurons during the active period (19:00 or ZT12). **(A)** *In vitro* confirmation. **(A1)** PZ^Vglut2–hM3Dq^ whole-cell recording showing an increase in firing frequency in response to bath application of CNO (0.5 μM). **(A2)** Average firing frequency (±S.E.M.) during the last 2 min of the CNO (0.5 μM) application as compared with the 2 min period preceding CNO application (control; *N* = 5 PZ^Vglut2–hM3Dq^ neurons). ^*^p < 0.05 Paired Student’s *t*-tests. **(B)** Extent of transduced neurons (mCherry-positive somas) is shown for individual *Vglut2-IRES-cre* mice that received bilateral injections of hM3Dq-mCherry-AAV into the PZ (PZ^Vglut2–hM3Dq^). **(C)** Hourly amount of wakefulness **(C1)**, SWS **(C2)** and REM sleep **(C3)** following CNO (0.3 mg/kg, *N* = 6 mice) as compared with control injection. **(D1–D3)** Percentage of sleep-wake states (±S.E.M.) during the 3 h post-injection period (19:00–22:00), the remainder (9 h) of the dark/active period (22:00–07:00) and the subsequent 12 h light period (07:00–19:00; *N* = 6 mice). **(E1–E3)** Number of episodes (±S.E.M.) of wakefulness (W), SWS or REM sleep (RS) in each bout length and **(E1’–E3’)** time-weighted frequency histograms showing the proportion (±S.E.M.) of W, SWS or RS amounts in each bout length as a percentage of the total amount of W, SWS or RS during the 3 h post-injection period (19:00–22:00; *N* = 6). **(F1–F3)** Sleep-wake power spectrum changes over baseline during the 3 h (19:00–22:00) post CNO (0.3 mg/kg, *N* = 4 mice) injection as compared with control injection; and the quantitative changes (±S.E.M.) in power for the δ (0.4–5 Hz), θ (5–9 Hz), α (9–15 Hz), β (15–30 Hz), low γ (30–60 Hz) and high γ (60–120 Hz) frequency bands (±S.E.M.) following vehicle or CNO (0.3 mg/kg, *N* = 4 mice) administrations. **(C–F)** Control injection in Black, CNO injection in red; ^*^p < 0.05, ^∗∗^*p* < 0.01, ^∗∗∗^*p* < 0.001, two-way ANOVA followed by a *post hoc* Bonferroni test.

For immunostaining, using a freezing microtome, brains were sectioned at 40 μm into 3 series. One series was used to label mCherry to visualize neurons transfected by hSyn-DIO-hM3Dq-mCherry-AAV. Floating brain sections were incubated overnight with the primary antiserum (1:10,000; rabbit polyclonal antibody against mCherry was raised against DsRed, catalog #632496, Clontech). The next day, sections were incubated in goat anti-rabbit biotinylated secondary antiserum (1:1,000; catalog # BA-1000, Vector Laboratories), followed by incubation in ABC reagents (1:1000; Vector Laboratories) for 90 min. Visualization reaction was in a 0.06% solution of 3,3-diaminobenzidine tetrahydrochloride (Sigma-Aldrich) in PBS plus 0.02% H_2_O_2_ for 2–15 min. Finally, the sections were mounted on slides, dehydrated, cleared, and coverslipped. To map the extent of hSyn-DIO-hM3Dq-mCherry-AAV transfection, immunostained neurons were visualized with a brightfield microscope (Keyence BZ-X710, Japan) and mapped (Figures 1B, 3A, 4A, 7A).

For RNAscope, using a cryostat (Thermo Scientific, Cryostar NX70), brains were sectioned at 10 μm and mounted onto Surgipath (Leica) adhesive microscope slides, 3 slices per slide. Slides were kept at −80°C until shortly before *in situ* hybridization. Slides were first warmed to room temperature and then we performed the RNAscope hybridization using a RNAscope Multiplex Fluorescent Reagent Kit (Advanced Cell Diagnostics, Inc., Newark, CA, United States). Briefly, according to the manufacturer’s instructions, target retrieval was performed at 99°C after which slices were dehydrated in 100% ethanol and air-dried. Next, sections were treated with protease inhibitor (Protease III, RNAscope) for 30 min at 40°C. After rinsing in RNAscope wash buffer, we incubated the sections in the RNAscope probes for Vglut2 (Mm-Slc17a6, catalog # 319171) and Phox2B (Mm-Phox2b-C2, catalog # 407861-C2). Additional sections were incubated in the manufacturer-supplied 2-plex positive control (catalog # 320761) and negative control (catalog # 320751) probes. Following the first 3 signal amplification steps, the fourth amplification was performed using Amp 4 Alt C-FL, such that channel 1 (Vglut2) was fluorescently labeled with Alto 550 and channel 2 (Phox2B) was labeled with Alto 647. Fluorescent images were collected with a confocal microscope (Zeiss LSM 700; Figures 6D–P).

Figures 6A–C, Phox2B-GFP Native GFP fluorescence images were collected with a fluorescence microscope (Keyence BZ-X710, Japan).

### Whole-Cell *in vitro* Experiments

For *in vitro* electrophysiological recordings, 10–13 days old Vglut2-ires-cre (*N* = 17) and Phox2B-ires-cre (*N* = 5) mice were injected bilaterally in the PZ, SLD or PB area with hSyn-DIO-hM3Dq-mCherry-AAV (100 nl/side). At about 3 weeks of age, 250 μm thick coronal brain slices of the PZ, SLD or PB area were prepared.

Mice were deeply anesthetized (200 mg/Kg Ketamine, 20 mg/Kg Xylazine) and transcardially perfused with ice-cold *N*-methyl-D-glucamine based artificial cerebrospinal fluid (NMDG-ACSF) containing (in mM): NMDG 98, HEPES 20, Glucose 25, NaHCO_3_ 30, Na-ascorbate 5, Na-pyruvate 3, Thiourea 2, MgSO_4_ 10, NaH_2_PO_4_ 1.24, KCl 2.5, CaCl_2_ 0.5; pH adjusted to ≈7.3 with HCl 37%. The brains were quickly extracted from the skull and sliced in carbogeneted (95% O2 5% CO2) ice-cold NMDG-ACSF using a vibrating microtome (7000-SMZ2, Campden Instruments). Slices containing the area of interest were immediately transferred to a chamber with carbogented NMDG-ACSF kept at 35°C for 8 min, then moved to carbogeneted normal ACSF at room temperature containing (in mM): NaCl 126, NaHCO_3_ 26, Glucose 10, Na-ascorbate 1, Thiourea 2, Na-Pyruvate 3, NaH_2_PO_4_ 1.24, KCl 2.5, CaCl_2_ 2, MgCl_2_ 1.3.

Recordings were guided using a combination of fluorescence and infrared differential interference contrast (IR-DIC) video microscopy using a fixed stage upright microscope (Axio Examiner.D1, Zeiss) equipped with a Nomarski immersion lens (40×/1.0) and an infrared-sensitive camera (Orca flash 4.0, Hamamatsu). Images were displayed in real time using Zen2 software (Carl Zeiss). Recordings were conducted in whole-cell mode using an EPC-10 USB amplifier and Patchmaster software (Heka).

Recordings were performed in current clamp mode using a K-gluconate based pipette solution containing (in mM): K-gluconate 120, KCl 10, MgCl_2_ 3, HEPES 10, K-ATP, Na-GTP 0.5. After at least 10 min of stable recording, ACSF containing CNO (500 nM) was perfused into the chamber for 3–5 min before washout. Recordings were analyzed, using Patchmaster software, by comparing the last 2 min before the application of CNO to the last 2 min of the CNO application. Paired Student’s *t*-tests were used to calculate statistical significance.

## Results

### PZ^Vglut2^ Neurons Are Not Sleep-Promoting

To test whether activation of PZ^Vglut2^ neurons affects sleep-wake phenotype, Vglut2-IRES-Cre mice were injected into the PZ with a virus vector containing the excitatory hM3Dq receptor (AAV-mCherry-hM3Dq) to specifically express hM3Dq receptors in PZ glutamatergic neurons (PZ^Vglut2–hM3Dq^ mice). First, responses of PZ^Vglut2–hM3Dq^ neurons to the hM3Dq ligand, clozapine-N-oxide (CNO), were tested using whole-cell *in vitro* recordings (Figure 1A1). Bath application of CNO (500 nM) significantly increased firing rates in PZ^Vglut2–hM3Dq^ neurons (1.2 ± 0.5 vs. 0.3 ± 0.2 Hz in control condition, *p* = 0.042; Figure 1A2), confirming that CNO activates PZ glutamatergic neurons. We then tested, *in vivo*, the sleep-wake phenotypes upon activation of PZ glutamatergic neurons. At the end of the behavioral studies, the injection sites were mapped using mCherry immunostaining. Of the sixteen Vglut2-IRES-Cre mice injected with AAV-mCherry-hM3Dq, six mice displayed more specific expression of mCherry bilaterally in the PZ (Figure 1B) and were used for the following sleep-wake analysis.

To test the effect of PZ^Vglut2^ neurons in sleep-wake control, mice were injected in a randomized cross-over design with saline or CNO (0.3 mg/kg) at the beginning of the dark/active period (19:00, ZT12) or during the light/rest period (10:00, ZT3). When injected at 19:00, CNO treatment did not significantly affect the hourly amount of wakefulness (two-way ANOVA, *F*(23,115) = 1.06, *p* = 0.40; Figure 1C1), SWS (two-way ANOVA, *F*(23,115) = 1.02, *p* = 0.45; Figure 1C2), or REM sleep (two-way ANOVA, *F*(23,115) = 1.38, *p* = 0.14; Figure 1C3). Because in previous studies (Anaclet et al., 2014, 2015, 2018) the effect of CNO-mediated neuronal activation or inhibition on sleep-wake cycles was most pronounced during the 3 h post-injection period, we performed a more refined analysis of this period. Neither wakefulness nor REM sleep amount and consolidation were affected by CNO injection during the 3 h post CNO injection as compared with control injection (Figures 1D1,D3,E1,E1’,E3,E3’). However, SWS amounts were significantly decreased during the 3 h post CNO injection period as compared with control injections (15.0 ± 5.0 vs. 33.5 ± 11.9% of SWS in control condition, *p* < 0.01; Figure 1D2) but without change in bout length distribution (Figure 1E2’). This SWS decrease is associated with a significant increase in the number of very short SWS bouts (7.0 ± 3.1 vs. 1.8 ± 0.6 bouts lasting between 10 and 30 s, *p* = 0.0006; Figure 1E2). Wakefulness bout duration was also increased with a significant increase of the proportion of wakefulness from long bout lengths (>40 min long bouts: 72.9 ± 10.1 vs. 33.3 ± 21.1% of total wakefulness after control injection, *p* = 0.003; Figure 1E1’). These results indicate more labile switching between the two vigilance stages. Cortical EEG power spectral distribution was affected by the treatment in wakefulness (two-way ANOVA, *F*(243,729) = 1.48, *p* < 0.0001; Figure 1F1) and SWS (two-way ANOVA, *F*(243,729) = 3.88, *p* < 0.0001; Figure 1F2) but not in REM sleep (two-way ANOVA, *F*(243,729) = 0.82, *p* = 0.97; Figure 1F3). However, none of the frequency bands displayed any significant difference between CNO and control injection, in any vigilance stage (Figures 1F1–F3). Altogether, activation of PZ glutamatergic neurons at a time when the wake-promoting systems are active, during the active phase, did not induce SWS and showed only minimal effects on sleep-wake phenotype, indicating that PZ^Vglut2^ neurons are not sleep-promoting.

**FIGURE 2:**
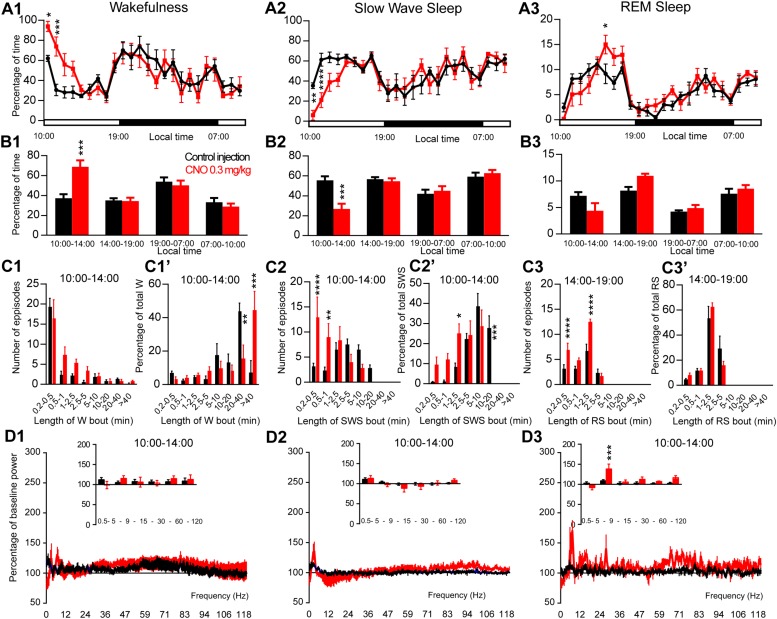
Activation of PZ glutamatergic neurons during the inactive period (10:00 or ZT3). **(A)** Hourly amount of wakefulness **(A1)**, SWS **(A2)** and REM sleep **(A3)** following CNO (0.3 mg/kg, *N* = 6 mice) as compared with control injection. **(B1–B3)** Percentage of sleep-wake states (±S.E.M.) during the 4 h post-injection period (10:00–14:00), the remainder (5 h) of the light/sleep period (14:00–19:00), the subsequent 12 h light period and first 3 h of the light period of the next day (07:00–10:00, *N* = 6 mice). **(C1–C3)** Number of episodes (±S.E.M.) of wakefulness (W), SWS or REM sleep (RS) in each bout length and **(C1’–C3’)** time-weighted frequency histograms showing the proportion (±S.E.M.) of W, SWS or RS amounts in each bout length as a percentage of the total amount of W, SWS or RS during the 4 h post-injection period (10:00–14:00; *N* = 6). **(D1–D3)** Sleep-wake power spectrum changes over baseline during the 3 h (10:00–13:00) post CNO (0.3 mg/kg, *N* = 5 mice) injection time period as compared with control injection; and the quantitative changes (±S.E.M.) in power for the δ (0.4–5 Hz), θ (5–9 Hz), α (9–15 Hz), β (15–30 Hz), low γ (30–60 Hz) and high γ (60–120 Hz) frequency bands (±S.E.M.) following vehicle or CNO (0.3 mg/kg, *N* = 5 mice) administrations. Control injection in Black, CNO injection in red; ^*^p < 0.05, ^∗∗^*p* < 0.01, ^∗∗∗^*p* < 0.001, ^*⁣*⁣**^p < 0.0001, two-way ANOVA followed by a *post hoc* Bonferroni test.

In order to test if activation of PZ^Vglut2^ neurons affects sleep-wake phenotypes differently when the sleep-promoting system is driving sleep, during the light period, CNO was injected at 10:00. CNO treatment significantly affected wakefulness (two-way ANOVA, *F*(23,115) = 3.14, *p* < 0.0001; Figure 2A1), SWS (two-way ANOVA, *F*(23,115) = 3.12, *p* < 0.0001; Figure 2A2) and REM sleep (two-way ANOVA, *F*(23,115) = 2.16, *p* = 0.004; Figure 2A3) hourly distribution. Wakefulness amount was significantly increased during the 4 h post CNO injection period (74.6 ± 7.5 vs. 40.3 ± 3.9% of time in control condition, *p* < 0.001; Figure 2B1). This wakefulness increase was at the expense of SWS (21.9 ± 6.1 vs. 53.5 ± 4.2% of time in control condition, *p* < 0.001; Figure 2B2). The increase in wakefulness was due to bout elongation, after CNO injection, as the mice were spending most of their wake time in bouts longer than 40 min (44.5 ± 11.2 vs. 7.2 ± 7.2% of total wakefulness after control injection, *p* < 0.001; Figure 2C1’), while in control condition, they were spending most of their wake time in 20–40 min long bouts (15.5 ± 8.2 vs. 44.3 ± 4.5% of total wakefulness in control condition, *p* = 0.01; Figure 2C1’). The number of wakefulness episodes, however, remained unchanged (Figure 2C1). The decrease of SWS was due to fragmentation characterized by a significant increase in the number of very short SWS bouts (13.0 ± 4.0 vs. 3.2 ± 0.6 episodes 30 s long or shorter in control condition, *p* < 0.0001; Figure 2C2) and a significant decrease of long SWS bouts (0.0 ± 0.0 vs. 27.8 ± 6.1% of total SWS in bouts 10–20 min long in control condition, *p* < 0.0001; Figure 2C2’). Interestingly, REM sleep amount displayed a trend to increase during the second part of the light period, 4 h following CNO injection (14:00–19:00; 10.3 ± 0.2 vs. 8.5 ± 0.7% of time after control injection, *p* > 0.05; Figure 2B3). This was associated with a significant increase in the number of episodes (10–30 s long bouts: 7.0 ± 1.3 vs. 3.3 ± 0.6 bouts after control injection, *p* < 0.0001; 10–30 s long bouts: 12.5 ± 0.6 vs. 6.8 ± 1.2 bouts after control injection, *p* < 0.0001; Figure 2C3). REM sleep bout length distribution, however, remained unchanged (Figure 2C3’). Cortical EEG power spectral distribution was not affected by the treatment in wakefulness (two-way ANOVA, *F*(243,972) = 0.76, *p* = 0.996; Figure 2D1). In contrast, both SWS (two-way ANOVA, *F*(243,972) = 2.79, *p* < 0.0001; Figure 2D2) and REM sleep (two-way ANOVA, *F*(243,972) = 1.43, *p* = 0.0001; Figure 2D3) cortical EEG power spectral distribution was affected by the treatment. Interestingly, during REM sleep, the theta band was significantly increased (128.1 ± 6.3 vs. 106.9 ± 3.2% of baseline theta power in control condition, *p* < 0.001; Figure 2D3).

### Activation of SLD^Vglut2^ Neurons During the Inactive Period Enhances REM Sleep

**FIGURE 3:**
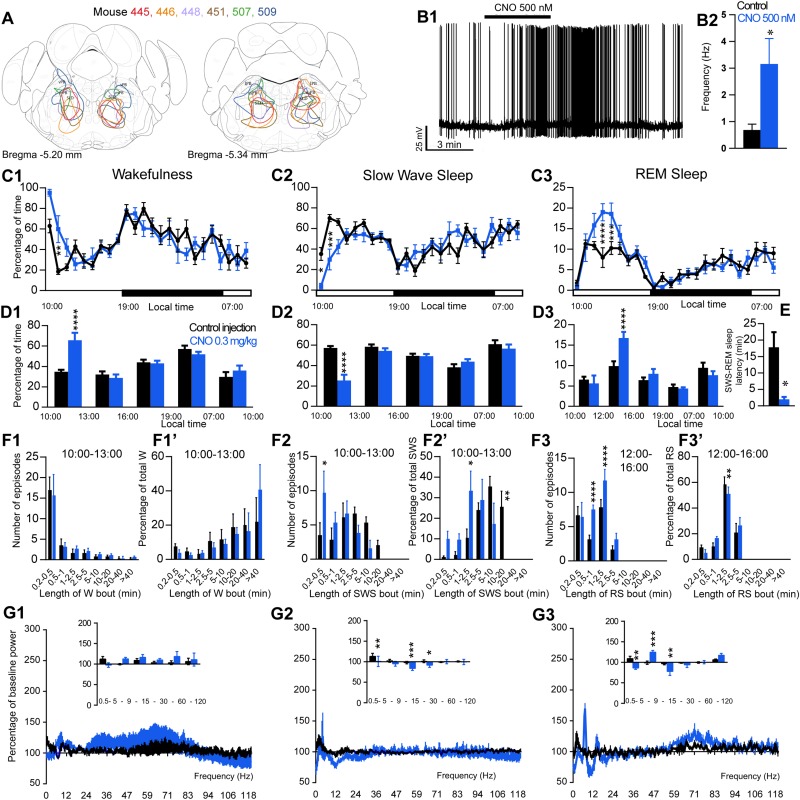
Activation of SLD glutamatergic neurons during the inactive period (10:00 or ZT3). **(A)** Extent of transduced neurons (mCherry-positive somas) is shown for individual *Vglut2-IRES-cre* mice that received bilateral injections of hM3Dq-mCherry-AAV into the SLD (SLD^Vglut2–hM3Dq^). **(B1)** SLD^Vglut2–hM3Dq^ whole-cell recording showing an increase in firing frequency in response to bath application of CNO (0.5 μM). **(B2)** Average firing frequency (±S.E.M.) during the last 2 min of the CNO (0.5 μM) application as compared with the 2 min period preceding CNO application (control; *N* = 6 PB^*rm Vglut2–hM3Dq*^ neurons). ^*^p < 0.05 Paired Student’s *t*-tests. **(C)** Hourly amount of wakefulness **(C1)**, SWS **(C2)** and REM sleep **(C3)** following CNO (0.3 mg/kg, *N* = 6 mice) as compared with control injection. **(D1,D2)** Percentage of wakefulness and SWS (±S.E.M.), respectively, during the 3 h post-injection period (10:00–13:00), the following 3 h (13:00–16:00), the remainder (3 h) of the light/sleep period (16:00–19:00), the subsequent 12 h dark period (19:00–07:00) and first 3 h of the light period of the next day (07:00–10:00, *N* = 6 mice). **(D3)** Percentage of REM sleep (±S.E.M.) during the 2 h post-injection period (10:00–12:00), the following 4 h (12:00–16:00), the remainder (3 h) of the light/sleep period (16:00–19:00), the subsequent 12 h dark period (19:00–07:00) and first 3 h of the light period of the next day (07:00–10:00, *N* = 6 mice). **(E)** SWS to REM sleep latency defined as the time (min) between the onset of the first SWS episode (>20 s) and the onset of the first REM sleep episode (>10 s). **(F1–F3)** Number of episodes (±S.E.M.) of wakefulness (W), SWS or REM sleep (RS) in each bout length and **(F1’–F3’)** time-weighted frequency histograms showing the proportion (±S.E.M.) of W, SWS or RS amounts in each bout length as a percentage of the total amount of W, SWS during the 3 h post-injection period (10:00–13:00) or RS during the 12:00–16:00 period (*N* = 6). **(G1–G3)** Sleep-wake power spectrum changes over baseline during the 3 h (10:00–13:00) post CNO (0.3 mg/kg, *N* = 6 mice) injection as compared with control injection; and the quantitative changes (±S.E.M.) in power for the δ (0.4–5 Hz), θ (5–9 Hz), α (9–15 Hz), β (15–30 Hz), low γ (30–60 Hz) and high γ (60–120 Hz) frequency bands (±S.E.M.) following vehicle or CNO (0.3 mg/kg, *N* = 6 mice) administrations. **(C–G)** Control injection in Black, CNO injection in blue; ^*^p < 0.05, ^∗∗^*p* < 0.01, ^∗∗∗^*p* < 0.001, ^*⁣*⁣**^p < 0.0001, two-way ANOVA followed by a *post hoc* Bonferroni test.

The excitatory receptor, hM3Dq, was mostly expressed in the SLD in six Vglut2-hM3Dq mice (SLD^Vglut2–hM3Dq^; Figure 3A). Whole-cell recording confirmed the expression of functional hM3Dq receptors (Figure 3B1). CNO (500 nM) application significantly increased the firing rate of SLD neurons (3.15 ± 0.96 vs. 0.68 ± 0.23 Hz in control condition, *p* = 0.032, Figure 3B2). Sleep-wake analysis during the inactive phase (10:00) revealed that CNO (0.3 mg/kg, *n* = 6, 10:00) injection significantly affected wakefulness (two-way ANOVA, *F*(23,115) = 2.38, *p* = 0.0014; Figure 3C1), SWS (two-way ANOVA, *F*(23,115) = 2.82, *p* = 0.0001; Figure 3C2) and REM sleep (two-way ANOVA, *F*(23,115) = 3.30, *p* < 0.0001; Figure 3C3) in SLD^Vglut2–hM3Dq^ mice, as compared with control injection. Wakefulness was significantly increased during the 3 h post CNO injection period (65.0 ± 8.8 vs. 34.8 ± 2.1% of time after control injection, *p* < 0.0001; Figure 3D1). At the same time, SWS amount was significantly decreased (25.9 ± 7.0 vs. 57.3 ± 1.8% of time after control injection, *p* < 0.0001; Figure 3D2). REM sleep amount remained unchanged during the 2 h post CNO injection period (6.2 ± 2.2 vs. 6.5 ± 0.7% of time after control injection, *p* > 0.05; Figure 3D3). However, REM sleep amount was significantly increased during the 2–6 h post-injection period (15.5 ± 1.1 vs. 9.8 ± 1.2% of time after control injection, *p* < 0.0001; Figure 3D3). Interestingly, the SWS to REM sleep latency was significantly decreased after CNO injection (2.0 ± 0.7 vs. 17.8 ± 4.6 min between the beginning of the first SWS episode and the beginning of the first REM sleep episode in control condition, *p* = 0.018; Figure 3E). The observed wakefulness increases during the 3 h post CNO injection resulted from a non-significant increase in both the number of long bouts (>40 min; Figure 3F1) and in the proportion of wakefulness from long bouts (>40 min; Figure 3F1’). SWS decrease was due to a significant decrease of the proportion of SWS from long SWS bouts (0.0 ± 0.0 vs. 26.0 ± 7.2% of total SWS from 10–20 min long bouts in control condition, *p* = 0.0038, Figure 3F2’), associated with a significant increase in the proportion of SWS from short SWS bouts (33.7 ± 9.2 vs. 10.8 ± 3.9% of total SWS from 1–2.5 min long bouts in control condition, *p* = 0.014, Figure 3F2’). At the same time, the number of very short SWS bouts (10–30 s long) were significantly increased (9.8 ± 3.0 vs. 3.5 ± 1.8 bouts in control condition, *p* = 0.015, Figure 3F2). The REM sleep increase 2–6 h post CNO injection was due to a significant increase in the number of medium-duration bouts (6.6 ± 1.6 vs. 3.6 ± 1.1 0.5–1 min long bouts in control condition, *p* < 0.0001; and 12.4 ± 3.0 vs. 6.8 ± 1.2 1–2.5 min long bouts in control condition, *p* < 0.0001, Figure 3F3) while REM sleep bout length is moderately affected (Figure 3F3’).

Cortical EEG power distribution was affected by CNO administration during wakefulness (two-way ANOVA, *F*(243,972) = 3.16, *p* < 0.0001; Figure 3G1), SWS (two-way ANOVA, *F*(243,972) = 2.27, *p* < 0.0001; Figure 3G2) and REM sleep (two-way ANOVA, *F*(243,972) = 4.89, *p* < 0.0001; Figure 3G3). During SWS, delta (101.1 ± 12.5 vs. 113.3 ± 7.4% of baseline power in control condition, *p* < 0.01), sigma (83.8 ± 4.7 vs. 98.0 ± 3.2% of baseline power in control condition, *p* < 0.001) and beta (91.9 ± 5.3 vs. 98.0 ± 3.2% of baseline power in control condition, *p* < 0.05) power bands were decreased (Figure 3G2). During REM sleep, both delta (86.5 ± 3.2 vs. 109.0 ± 5.9% of baseline power in control condition, *p* < 0.01) and sigma (78.1 ± 10.1 vs. 96.8 ± 3.0% of baseline power in control condition, *p* < 0.01) frequency bands were significantly decreased whereas theta (125.0 ± 3.9 vs. 99.1 ± 3.9% of baseline power in control condition, *p* < 0.001) was significantly increased (Figure 3G3). Similar to PZ^Vglut2–hM3Dq^ mice, SLD^Vglut2–hM3Dq^ did not show any sleep-wake phenotypes when CNO was injected at the beginning of the dark/active period (data not shown), indicating a time of the day difference.

### Activation of PB^Vglut2^ Neurons Induces Wakefulness

**FIGURE 4:**
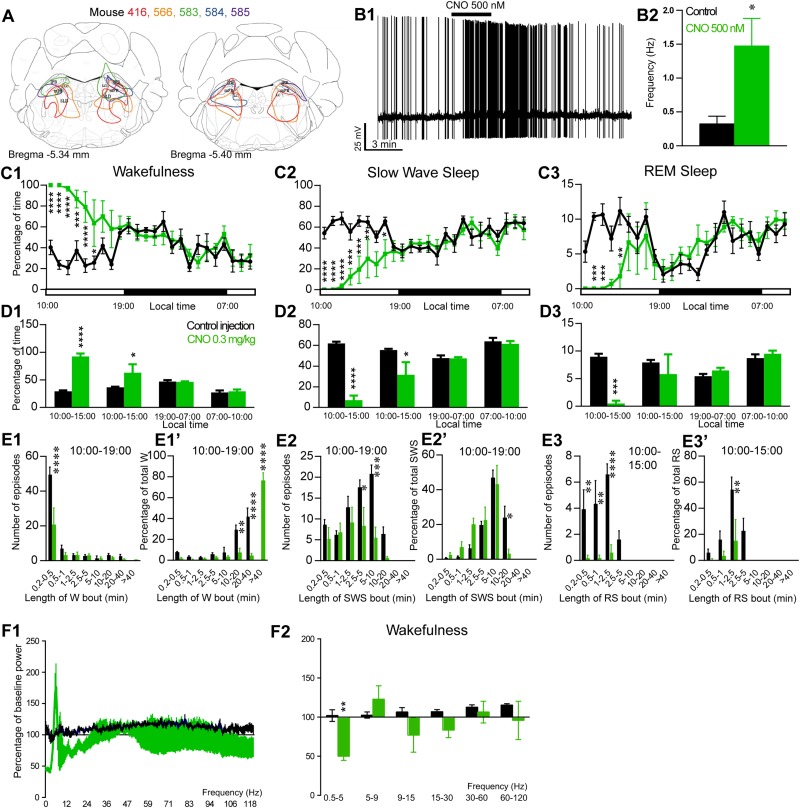
Activation of PB glutamatergic neurons during the inactive period (10:00 or ZT3). **(A)** Extent of transduced neurons (mCherry-positive somas) is shown for individual *Vglut2-IRES-cre* mice that received bilateral injections of hM3Dq-mCherry-AAV into the PB (PB^Vglut2–hM3Dq^). **(B1)** PB^Vglut2–hM3Dq^ whole-cell recording showing an increase in firing frequency in response to bath application of CNO (0.5 μM). **(B2)** Average firing frequency (±S.E.M.) during the last 2 min of CNO (0.5 μM) application as compared with the 2 min period preceding CNO application (control; *N* = 6 PB^Vglut2–hM3Dq^ neurons). ^*^p < 0.05 Paired Student’s *t*-tests. **(C)** Hourly amount of wakefulness **(C1)**, SWS **(C2)** and REM **(C3)** sleep following CNO (0.3 mg/kg, *N* = 5 mice) as compared with control injection. **(D1–D3)** Percentage of sleep-wake states (±S.E.M.) during the 5 h post-injection period (10:00–15:00), the following 4 h of the light period (15:00–19:00), the subsequent 12 h dark/wake period (19:00–07:00) and first 3 h of the light period of the next day (07:00–10:00, *N* = 5 mice). **(E1,E2)** Number of episodes (±S.E.M.) of wakefulness (W) or SWS in each bout length and **(E1’,E2’)** time-weighted frequency histograms showing the proportion (±S.E.M.) of W or SWS amounts in each bout length as a percentage of the total amount of W or SWS during the 9 h post-injection period (10:00–19:00; *N* = 6). **(E3)** Number of episodes (±S.E.M.) of REM sleep (RS) in each bout length and **(E3’)** time-weighted frequency histograms showing the proportion (±S.E.M.) of RS amounts in each bout length as a percentage of the total amount of W or SWS during the 5 h post-injection period (10:00–15:00; *N* = 6). **(F1)**Wake power spectrum changes over baseline during the 3 h (10:00–13:00) post CNO (0.3 mg/kg, *N* = 5 mice) injection period as compared with control injection. **(F2)** Quantitative changes (±S.E.M.) in power for the δ (0.4–5 Hz), θ (5–9 Hz), α (9–15 Hz), β (15–30 Hz), low γ (30–60 Hz) and high γ (60–120 Hz) frequency bands (±S.E.M.) following vehicle or CNO (0.3 mg/kg, *N* = 5 mice) administrations. Power spectral analysis was not performed for slow-wave-sleep and REM sleep due to the low amount of these vigilance stages. **(C–F)** Control injection in Black, CNO injection in green; ^*^p < 0.05, ^∗∗^*p* < 0.01, ^∗∗∗^*p* < 0.001, ^*⁣*⁣**^p < 0.0001, two-way ANOVA followed by a *post hoc* Bonferroni test.

The excitatory receptor, hM3Dq, was mostly expressed in the PB in five Vglut2-hM3Dq mice (PB^Vglut2–hM3Dq^; Figure 4A). Slice electrophysiology showed that firing rates of PB^Vglut2–hM3Dq^ neurons were significantly increased (1.48 ± 0.40 vs. 0.32 ± 0.04 Hz in control condition, *p* = 0.042) by bath application of CNO (500 nM; Figures 4B1,B2). Injection of CNO (0.3 mg/kg, 10:00) in PB^Vglut2–hM3Dq^ mice significantly affected wakefulness (two-way ANOVA, *F*(23,92) = 6.12, *p* < 0.0001; Figure 4C1), SWS (two-way ANOVA, *F*(23,92) = 6.24, *p* < 0.0001; Figure 4C2) and REM sleep (two-way ANOVA, *F*(23,92) = 3.44, *p* < 0.0001; Figure 4C3). Wakefulness amount was significantly increased during the remaining 9 h of the light period post-injection (79.3 ± 9.5 vs. 32.4 ± 1.5% of time, *p* < 0.001; Figure 4D1). At the same time, both SWS (17.8 ± 7.8 vs. 59.1 ± 1.4% of time, *p* < 0.001; Figures 4C2–D3) and REM sleep (2.9 ± 1.7 vs. 8.5 ± 0.4% of time, *p* < 0.01; Figures 4C3–D3) amount were significantly decreased. No sleep rebound followed the long-lasting wakefulness increase (47.5 ± 1.1 vs. 47.7 ± 2.7% of time spent in SWS during the following dark period, 19:00–07:00, *p* > 0.05; Figures 4C2,D2). Wakefulness enhancement was due to a significant increase in bout length (76.7 ± 7.3 vs. 0.0 ± 0.05 of wakefulness from >40 min long bouts, *p* < 0.0001, Figure 4E1’), associated with a significant decrease in the number of short episodes (Figure 4E1). Both SWS bout number (5.6 ± 2.5 vs. 20.8 ± 2.1 5–10 min long bouts, *p* = 0.0002; Figure 4E2) and bout duration (3.4 ± 2.4 vs. 23.9 ± 6.6% of SWS in 10–20 min long bouts, *p* = 0.025; Figure 4E2’) were significantly decreased. Similarly, both REM sleep bout number (Figure 4E3) and bout duration (Figure 4E3’) were significantly decreased during the 5 h period following injection. Cortical EEG power distribution was affected by CNO injection during wakefulness (two-way ANOVA, *F*(543,972) = 1.66, *p* < 0.0001; Figure 4F1). PB^Vglut2^ induced wakefulness was characterized by a significant decrease in cortical EEG delta power (49.9 ± 5.2 vs. 102.1 ± 7.4% of baseline power in control condition, *p* < 0.01; Figure 4F2). Similar results were obtained when CNO was injected at the beginning of the active period (19:00; not shown).

### CNO Does Not Affect Sleep-Wake Cycle in Control Mice

**FIGURE 5:**
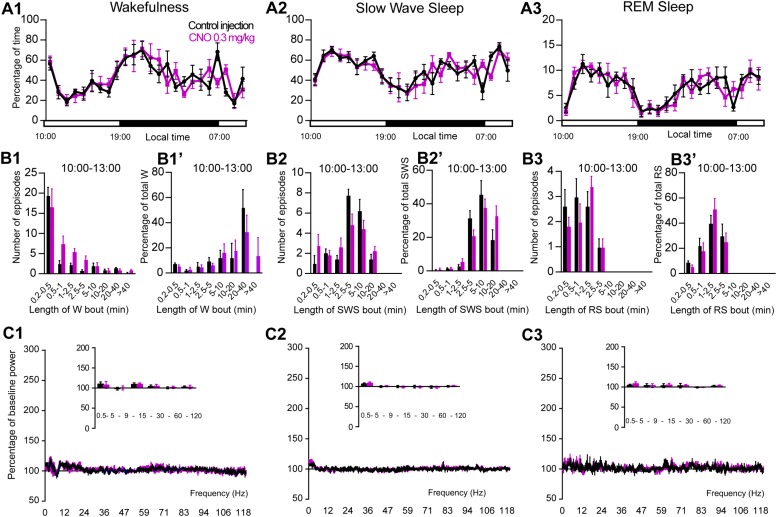
CNO injection (10:00 or ZT3) in control mice. **(A)** Hourly amount of wakefulness **(A1)**, SWS **(A2)** and REM **(A3)** sleep following CNO (0.3 mg/kg, *N* = 5 mice) as compared with control injection. **(B1–B3)** Number of episodes (±S.E.M.) of wakefulness (W), SWS or REM sleep (RS) in each bout length and **(B1’–B3’)** time-weighted frequency histograms showing the proportion (±S.E.M.) of W, SWS or RS amounts in each bout length as a percentage of the total amount of W, SWS or RS during the 3 h post-injection period (10:00–13:00; *N* = 5). **(C1–C3)** Sleep-wake power spectrum changes over baseline during the 3 h (10:00–13:00) post CNO (0.3 mg/kg, *N* = 5 mice) injection as compared with control injection; and the quantitative changes (±S.E.M.) in power for the δ (0.4–5 Hz), θ (5–9 Hz), α (9–15 Hz), β (15–30 Hz), low γ (30–60 Hz) and high γ (60–120 Hz) frequency bands (±S.E.M.) following vehicle or CNO (0.3 mg/kg, *N* = 5 mice) administrations. Control injection in Black, CNO injection in magenta; no significant change, two-way ANOVA followed by a *post hoc* Bonferroni test.

To control for non-specific actions of CNO, non-cre expressing littermate mice were used. No hM3Dq receptor transfection was seen in these control mice. Treatment did not affect the hourly distribution of wakefulness (two-way ANOVA, *F*(23,92) = 1.53, *p* = 0.082; Figure 5A1), SWS (two-way ANOVA, *F*(23,92) = 1.56, *p* = 0.071; Figure 5A2) or REM sleep (two-way ANOVA, *F*(23,92) = 1.06, *p* = 0.4; Figure 5A3). Moreover, CNO treatment did not affect the number of episodes or the episode length distribution as compared with control injection (Figures 5B1–B3’) in any vigilance state. Finally, the cortical EEG power distribution during wakefulness, SWS and REM sleep was similar after CNO injection, as compared with both control injection and baseline recording (Figures 5C1–C3). These results confirm that the sleep-wake effects seen in PZ^Vglut2–hM3Dq^, PB^Vglut2–hM3Dq^ and SLD^Vglut2–hM3Dq^ mice is due to the specific activation of glutamatergic neurons.

### Phox2B Is a Specific Marker for PZ Glutamatergic Neurons

**FIGURE 6:**
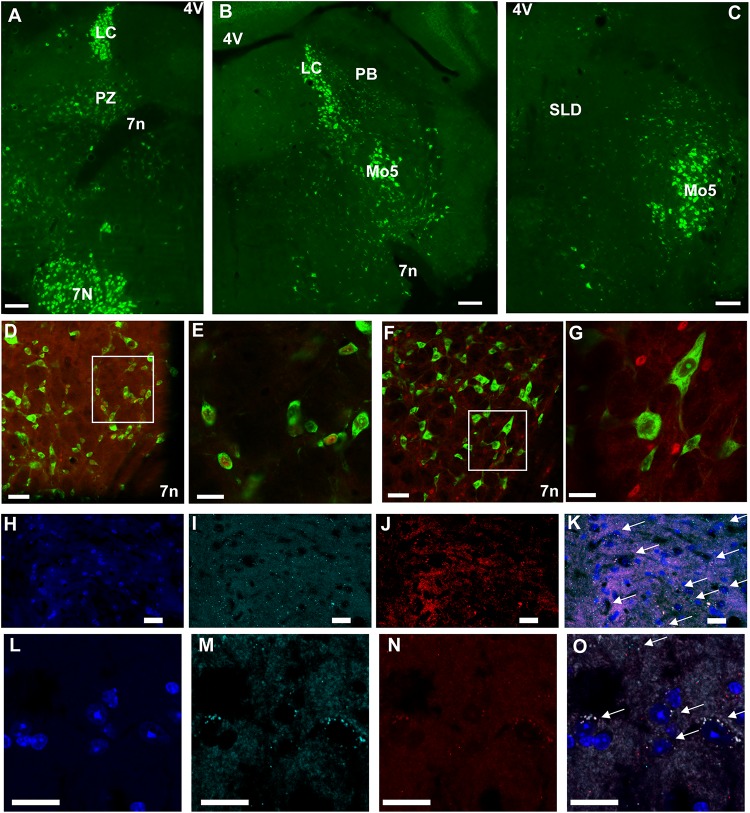
Phox2B is expressed in PZ and is co-localized with Vglut2. **(A–C)** Images from a Phox2B-GFP mouse brain showing Phox2B-expressing neurons in PZ **(A)** but not in PB **(B)** or SLD **(C)**. Scale bar: 200 μm. **(D)** Confocal images from PZ showing native GFP from a Vglut2-GFP mouse immunolabeled for Phox2B in red. Scale bar: 50 μm. **(E)** Higher magnification of the box in **(D)**. Scale bar: 20 μm. **(F)** Confocal images from PZ showing native GFP from a Vgat-GFP mouse immunolabeled for Phox2B in red. Scale bar: 50 μm. **(G)** higher magnification of the box in **(F)**. Scale bar: 20 μm. **(H–O)** Confocal images of the PZ showing DAPI staining of nuclei **(H,L)**, and mRNA of Vglut2 **(I,M)** and Phox2B **(J,N)** from two mice. The merged images show complete overlap of both mRNA expression (arrows in **K**,**O**). Scale bar: 20 μm. Abbreviations: 4 V, fourth ventricle; 7 n, seventh facial nerve; LC, locus coeruleus; Mo5, motor trigeminal nucleus; PB, parabrachial nucleus; PZ, parafacial zone; SLD, sublaterodorsal nucleus.

Because chemogenetic activation of PZ^Vglut2^ neurons resulted in phenotypes resembling chemogenetic activation of PB^Vglut2^ and SLD^Vglut2^ neurons, i.e., wakefulness and REM sleep increase, respectively, we hypothesized that in the PZ^Vglut2–hM3Dq^ mouse group some PB^Vglut2^ and SLD^Vglut2^ neurons were transfected and therefore responsible for the phenotypes. To specifically target PZ glutamatergic neurons we took advantage of a specific marker for PZ glutamatergic neurons, Phox2B. In the adult rat medullary and pontine regions in proximity to the PZ, Phox2B expression is restricted to the PZ, with a notable lack of expression in either the PB or the SLD (Kang et al., 2007). Moreover, Phox2B is co-localized with Vglut2 but not with Vgat or GAD67 [Figures 6D–G; (Stornetta et al., 2006)], suggesting that Phox2B is a specific marker for PZ glutamatergic neurons. We first confirmed the presence of Phox2B expression in PZ (Figure 6A) of mouse using Phox2B-GFP mice. No GFP positive neurons were seen in either the PB or the SLD (Figures 6B,C), indicating that Phox2B is specific for PZ glutamatergic neurons. Neurons of the locus coeruleus were GFP positive (LC; Figures 6A,B), which is consistent with previous studies showing that Phox2B is necessary for the differentiation of central noradrenergic and adrenergic neurons (Pattyn et al., 2000; Huber et al., 2005). We then assessed the extent of co-localization between Vglut2 and Phox2B in PZ. In each of the slices containing the PZ (*n* = 12 from 4 mice), Vglut2 co-localized exclusively with Phox2B and Phox2B was found primarily lateral to the facial nerve, in the entire PZ area (Figures 6H–K). Higher magnification photomicrographs show the cellular details of Vglut2/Phox2B co-localization Figures 6L–O). Therefore, Phox2B is a specific marker for PZ glutamatergic neurons and Phox2B-IRES-cre mice can be used to specifically activate PZ glutamatergic neurons and study their role in sleep-wake control.

### Activation of PZ^Phox2B^ Neurons Does Not Affect Sleep-Wake Cycle

**FIGURE 7:**
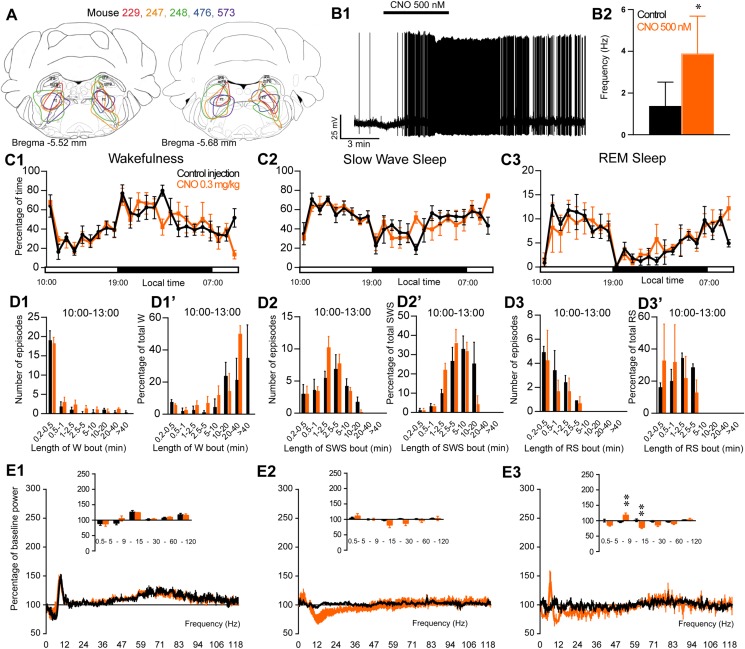
Activation of PZ Phox2B-expressing neurons during the inactive period (10:00 or ZT3). **(A)** Extent of transduced neurons (mCherry-positive somas) is shown for individual *Phox2B-IRES-cre* mice that received bilateral injections of hM3Dq-mCherry-AAV into the PZ (PZ^Phox2B–hM3Dq^). **(B1)** PZ^Phox2B–hM3Dq^ whole-cell recording showing an increase in firing frequency in response to bath application of CNO (0.5 μM). **(B2)** Average firing frequency (±S.E.M.) during the last 2 min of CNO (0.5 μM) application as compared with the 2 min period preceding CNO application (control; *N* = 5 PZ^Phox2B–hM3Dq^ neurons). ^*^p < 0.05 Paired Student’s *t*-tests. **(C)** Hourly amount of wakefulness **(C1)**, SWS **(C2)** and REM sleep **(C3)** following CNO (0.3 mg/kg, 10 A.M., *N* = 4 mice) as compared with control injection. **(D1–D3)** Number of episodes (±S.E.M.) of wakefulness (W), SWS, or REM sleep (RS) in each bout length and **(D1’–D3’)** time-weighted frequency histograms showing the proportion (±S.E.M.) of W, SWS or RS amounts in each bout length as a percentage of the total amount of W, SWS or RS during the 3 h post-injection period (10:00–19:00; *N* = 4). **(E1–E3)** Sleep-wake power spectrum changes over baseline during the 3 h (10:00–13:00) post CNO (0.3 mg/kg, *N* = 4 mice) injection as compared with control injection; and the quantitative changes (±S.E.M.) in power for the δ (0.4–5 Hz), θ (5–9 Hz), α (9–15 Hz), β (15–30 Hz), low γ (30–60 Hz) and high γ (60–120 Hz) frequency bands (±S.E.M.) following vehicle or CNO (0.3 mg/kg, *N* = 4 mice) administrations. **(C–E)** Control injection in Black, CNO injection in orange; ^∗∗^*p* < 0.01, two-way ANOVA followed by a *post hoc* Bonferroni test.

To assess the involvement of PZ glutamatergic neurons, five Phox2B-IRES-Cre mice were injected into the PZ with AAV-hM3Dq-mCherry (Figure 7A). Three of the five cases also showed partial expression in the LC. Whole-cell *in vitro* recording (Figure 7B1) confirmed that bath application of CNO (500 nM) significantly increased firing rate in PZ^Phox2B–hM3Dq^ neurons (3.9 ± 1.8 vs. 1.4 ± 1.2 Hz in control condition, *p* = 0.028; Figure 7B2). CNO injection was successful in four of the five PZ^Phox2B–hM3Dq^ mice (one mouse displayed an atypical adverse reaction to the injection). CNO treatment during the light period (10:00) did not affect the hourly amounts of wakefulness (two-way ANOVA, *F*(23,69) = 1.26, *p* = 0.23; Figure 7C1), SWS (two-way ANOVA, *F*(23,69) = 1.21, *p* = 0.27; Figure 7C2) or REM sleep (two-way ANOVA, *F*(23,69) = 0.92, *p* = 0.57; Figure 7C3). In order to study the qualitative aspects of the sleep-wake cycle following activation of PZ^Phox2B^ neurons, we studied fragmentation (Figures 7D1–D3’) and cortical EEG power distribution (Figures 7E1–E3) of the three vigilance stages during the 3 h post-injection time period. The number of sleep-wake episodes and episode length distribution were similar between CNO and control injections (Figures 7D1–D3). Treatment did not affect the cortical EEG power distribution during wakefulness and SWS. However, during REM sleep, the theta frequency band was significantly increased (118.4 ± 6.8 vs. 96.5 ± 2.5% of baseline power in control condition, *p* < 0.01; Figure 7D3) while the sigma frequency band was significantly decreased (78.4 ± 3.8 vs. 101.6 2.5% of baseline power in control condition, *p* < 0.01; Figure 7D3). These results indicate that activation of PZ^Phox2B–hM3Dq^ during the light, inactive, period does not affect the sleep-wake cycle but could be involved in cortical EEG activation during REM sleep. Similar results were obtained when CNO was administrated at the beginning of the active period (19:00; Figure 8).

**FIGURE 8:**
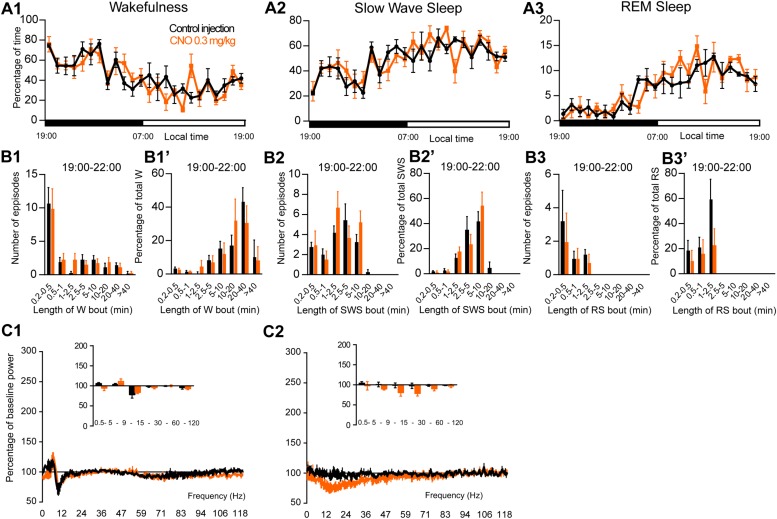
Activation of PZ Phox2B-expressing neurons during the active period (19:00 or ZT12). **(A)** Hourly amount of wakefulness **(A1)**, SWS **(A2)** and REM sleep **(A3)** following CNO (0.3 mg/kg, *N* = 4 mice) as compared with control injection. **(B1–B3)** Number of episodes (±S.E.M.) of wakefulness (W), SWS or REM sleep (RS) in each bout length and **(B1’–B3’)** time-weighted frequency histograms showing the proportion (±S.E.M.) of W, SWS or RS amounts in each bout length as a percentage of the total amount of W, SWS or RS during the 3 h post-injection period (19:00–22:00; *N* = 4). **(C1–C3)** Sleep-wake power spectrum changes over baseline during the 3 h (19:00–22:00) post CNO (0.3 mg/kg, *N* = 3 mice) injection as compared with control injection; and the quantitative changes (±S.E.M.) in power for the δ (0.4–5 Hz), θ (5–9 Hz), α (9–15 Hz), β (15–30 Hz), low γ (30–60 Hz) and high γ (60–120 Hz) frequency bands (±S.E.M.) following vehicle or CNO (0.3 mg/kg, *N* = 3 mice) administrations. Power spectral analysis was not performed for REM sleep due to the low amount of this vigilance stage. Control injection in Black, CNO injection in orange; no significant change, two-way ANOVA followed by a *post hoc* Bonferroni test.

## Discussion

To test the contribution of PZ glutamatergic neurons in sleep-wake control, we chemogenetically activated Vglut2-expressing neurons. Based on the sleep-wake phenotype and anatomical confirmation of the injection sites, the mice were separated in three groups: (1) one group, with targeted neuronal transfection mainly in the PZ, displayed increased wakefulness followed by a trend to increased REM sleep during the rest period but not during the active period; (2) a second group, with transfections that included the SLD, displayed a shorter wake increase followed by a significant increase in REM sleep amount; and (3) the third group, which had significant PB transfection, displayed a prominent and long lasting increase in wakefulness, independent of the time of day the injections were performed. Thus, due to the difficulty in targeting only glutamatergic PZ neurons while avoiding glutamatergic neurons in surrounding areas, the role of PZ^Vglut2^ neurons in sleep-wake control was still unclear. CNO did not affect the sleep-wake cycle in control mice not expressing the hM3Dq receptor, confirming that the phenotypes seen in Vglut2 transfected mice were specifically due to the activation of glutamatergic neurons. To test the specific role of PZ glutamatergic neurons in sleep-wake control, we took advantage of Phox2B, a transcription factor expressed by a subset of brainstem glutamatergic neurons. We first confirmed that Phox2B is a specific marker for PZ glutamatergic neurons in mice. Because chemogenetic activation of PZ^Phox2B^ neurons did not affect sleep-wake phenotypes, we can conclude that PZ glutamatergic neurons are not sufficient to influence the sleep-wake cycle.

The absence of a sleep-wake phenotype in control and PZ^Phox2B–hM3Dq^ mice after CNO injection provides additional evidence that CNO, at the dose used in our studies, does not affect the baseline sleep-wake cycle. A recent study had shown that clozapine, a metabolite of CNO, but not CNO, crosses the blood brain barrier and binds chemogenetic receptors with high affinity in rats (Gomez et al., 2017). This finding was subsequently challenged by the observation that both clozapine and CNO cross the blood brain barrier in mice, and that unbound CNO is present in the brain at concentrations sufficient to activate DREADDs, albeit at a higher initial dose than we typically use (Jendryka et al., 2019). We had previously shown that, at a dose of 0.3 mg/kg, CNO does not affect sleep-wake quantity and quality in Vgat-IRES-cre mice (Anaclet et al., 2014), nor does it interfere with the wake-promoting actions of armodafinil and caffeine (Anaclet et al., 2018). In the present study, we confirm the absence of non-specific actions of CNO on the sleep-wake cycle, using two different mouse strains, Vglut2-IRES-cre and Phox2B-IRES-cre mice. Additionally, we confirmed that CNO is able to directly activate PZ^Vglut2–hM3Dq^, PB^Vglut2–hM3Dq^, and SLD^Vglut2–hM3Dq^ neurons *in vitro*, where the short application duration (few minutes) and the absence of hepatic metabolism make back-conversion to clozapine highly unlikely. In summary, CNO was able to activate glutamatergic neurons expressing hM3Dq chemogenetic receptor and did not result in non-specific sleep-wake phenotypes.

### Phox2B Is a Specific Marker for PZ Glutamatergic Neurons

The transcription factor Phox2B has been studied for its involvement in the control of breathing and autonomic regulation. Phox2B mutations have been implicated in congenital central hypoventilation syndrome (Moreira et al., 2016). Phox2B-expressing neurons located in the medullary retrotrapezoid nucleus (RTN), ventral from the facial nucleus, are sensitive to hypoxia (Onimaru et al., 2008), hypercapnic acidosis and serotonin (Wu et al., 2019). Phox2B is necessary for the differentiation of central noradrenergic and adrenergic neurons (Pattyn et al., 2000; Huber et al., 2005). Phox2B is expressed in PZ in adult rats (Kang et al., 2007) but these neurons have no known physiological function. In the present study, we showed that, in PZ, Phox2B is highly co-localized with Vglut2 and therefore, is a specific marker for PZ glutamatergic neurons. Phox2B is also highly co-localized with LC noradrenergic neurons, known to be wake-promoting. However, in this study, the three mice showing partial expression of hM3Dq receptors in LC, did not display an increase in wake amount following CNO injection. It is possible that either the partial coverage of LC was not enough to promote wakefulness or LC noradrenergic neurons were not activated by the chemogenetic ligand. *In vitro* recordings of LC^Phox2B–hM3Dq^ neurons would be necessary to answer this question.

### The Role of PZ Glutamatergic Neurons in Sleep-Wake Control

A previous study has suggested that some PZ non-GABAergic neurons are sleep-active (Anaclet et al., 2012). Because glutamatergic neurons are the only other neuronal population identified in PZ thus far, we tested if chemogenetic activation of PZ glutamatergic neurons affects sleep-wake phenotypes. Specific targeting of PZ glutamatergic neurons using Vglut2-cre mice was challenging. Of the over 29 injected mice, only six displayed hM3Dq expression mainly in PZ (PZ^Vglut2–hM3Dq^). Five mice displayed hM3Dq expression mainly in PB (PB^Vglut2–hM3Dq^) and six in SLD (SLD^Vglut2–hM3Dq^). The remaining mice included seven showing expression of hM3Dq at multiple sites, and five died after surgery or during the sleep recordings. These last two mouse groups were excluded from the study.

Chemogenetic activation of PZ^Vglut2–hM3Dq^ neurons at the beginning of the mouse active phase (19:00) had limited impact on the sleep-wake cycle. On the other hand, chemogenetic activation of PZ^Vglut2–hM3Dq^ neurons during the mouse rest phase (10:00) resulted in an early wake enhancement followed by an increase in REM sleep amount. Because these phenotypes are reminiscent of the phenotypes observed in PB^Vglut2–hM3Dq^ and SLD^Vglut2–hM3Dq^ mice, we hypothesized that they were due to the inadvertent transfection of PB and SLD neurons. In other words, in the PZ^Vglut2–hM3Dq^ group, transfection would not be restricted to PZ. To test this hypothesis and definitively confirm the role of PZ^Vglut2^ neurons in sleep-wake control, we took advantage of Phox2B, a specific marker for PZ^Vglut2^ neurons. Using Phox2B-cre mice to specifically target PZ^Vglut2^ neurons and not neighboring PB and SLD, we showed that PZ^Vglut2^ neurons are not sufficient to affect the sleep-wake cycle at any time of the day. These results indicate that PZ glutamatergic neurons have no role in sleep or wake induction and/or maintenance. It remains, however, to be tested whether PZ^Vglut2^ neurons are necessary for normal sleep-wake cycle control, using inhibitory chemogenetic receptors and/or cell body specific lesion.

### A New Mouse Model for REM Sleep Enhancement

Rostral to the PZ and PB, the SLD contains Vglut2-expressing neurons that are specifically active during REM sleep recovery (Clement et al., 2011). The SLD contains a large proportion of neurons with tonic discharge patterns immediately prior to and during REM sleep (Sakai, 2015). Cell body specific SLD lesions, knockout of glutamatergic transmission and genetic inactivation significantly reduce REM sleep amount and result in REM sleep without muscle atonia (Lu et al., 2006; Krenzer et al., 2011; Valencia Garcia et al., 2017). In the present study, we show for the first time that chemogenetic activation of SLD^Vglut2^ neurons results in increased REM sleep amount and reduced SWS to REM sleep latency. Moreover, cortical EEG theta power is significantly enhanced during REM sleep. These data provide a new and unique model of REM sleep enhancement. Such a model will permit probing of the specific role of REM sleep in other neurophysiological functions, such as memory consolidation. However, specific targeting of SLD glutamatergic neurons is challenging due to the close proximity of PB wake-promoting glutamatergic neurons (Fuller et al., 2011). A specific marker for SLD glutamatergic neurons would be very useful.

### Additional Evidence for the Importance of PB in Wakefulness

In close proximity to PZ, just dorsal, lateral and rostral, the PB is a critical brainstem wake-promoting system. Following lesions of both PB and precoeruleus (PC), rats can no longer sustain cortical activation and become comatose (Fuller et al., 2011). Since this seminal study, the role of PB glutamatergic neurons in wakefulness has been refined. Specific lesions of medial PB result in hypersomnolence (Kaur et al., 2013). Glutamatergic neurons located in the external lateral PB are activated by hypoxia and are a key component of the vitally important circuitry regulating arousal from sleep apnea episodes (Kaur et al., 2017). In the present study we show that chemogenetic activation of medial PB results in long lasting wake enhancement. Moreover, CNO induced wakefulness was characterized by a decreased delta frequency band power. Because the delta band is considered a marker of EEG synchronization and is more prominent during quiet wakefulness, this result indicates a more active wake state induced by activation of PB^Vglut2^ neurons. Finally, no sleep rebound was seen after the wake enhancement. This is in accordance with previous studies using chemogenetics to specifically activate wake-promoting neuronal populations (Anaclet et al., 2015; Venner et al., 2016; Pedersen et al., 2017) and indicates that chemogenetic activation of wake-promoting neuronal populations does not enhance the homeostatic drive for sleep. All together, these results confirm the strong wake-promoting action of PB glutamatergic neurons.

## Conclusion

This study shows, for the first time, that PZ glutamatergic neurons are not sufficient to affect the sleep-wake cycle in mouse. However, chemogenetic activation of PB or SLD glutamatergic neurons results in wake or REM sleep enhancement, respectively. Finally, Phox2B is a specific marker for PZ glutamatergic neurons. All together, these results provide a better understanding on how the brain regulates sleep-wake cycles, forming a framework for future studies characterizing the sleep-promoting subpopulation of the PZ.

## Data Availability

The raw data supporting the conclusions of this manuscript will be made available by the authors, without undue reservation, to any qualified researcher.

## Ethics Statement

All procedures were approved by the Institutional Animal Care and Use Committee of Beth Israel Deaconess Medical Center and of University of Massachusetts Medical School.

## Author Contributions

EE performed the immunostaining and analyzed the sleep data. LF performed and analyzed the *in vitro* experiments. HG performed and analyzed the *in situ* hybridization experiments and wrote the manuscript. CA performed the surgeries and the *in vivo* experiments, and wrote the manuscript.

## Conflict of Interest Statement

The authors declare that the research was conducted in the absence of any commercial or financial relationships that could be construed as a potential conflict of interest.

## Footnotes

1 http://mouse.brain-map.org/experiment/show/73818754
